# Effects of vaspin on pancreatic β cell secretion via PI3K/Akt and NF-κB signaling pathways

**DOI:** 10.1371/journal.pone.0189722

**Published:** 2017-12-14

**Authors:** Shiwei Liu, Xin Li, Yaru Wu, Ruixue Duan, Jiaxin Zhang, Fang Du, Qi Zhang, Yuanbin Li, Naishi Li

**Affiliations:** 1 Department of Endocrinology, Shanxi DAYI Hospital, Shanxi Medical University, Taiyuan, China; 2 Central Laboratory, Taiyuan Central Hospital, Shanxi Medical University, Taiyuan, China; 3 Graduate School of Shanxi Medical University, Taiyuan, China; 4 Department of Endocrinology, Taiyuan Central Hospital, Shanxi Medical University, Taiyuan, China; 5 Department of Endocrinology, Key Laboratory of Endocrinology, Peking Union Medical College Hospital, Chinese Academy of Medical Science, Beijing, China; University of Michigan, UNITED STATES

## Abstract

Vaspin (visceral adipose tissue-derived serine protease inhibitor) is a recently discovered adipokine that has been implicated in diabetes mellitus and other metabolic disorders. However, the effects of vaspin on pancreatic β cell function and related mechanisms are not fully understood. Thus, the present study was performed to investigate the effects of vaspin on pancreatic β cell function and the potential underlying mechanisms. Both in vitro (rat insulinoma cells, INS-1) and in vivo (high fat diet fed rats) experiments were conducted. The results showed that vaspin significantly increased INS-1 cell secretory function. Potential mechanisms were explored using inhibitors, western blot and real-time PCR techniques. We found that vaspin increased the levels of IRS-2 mRNA and IRS-2 total protein, while decreased the serine phosphorylation level of IRS-2 protein. Moreover, vaspin increased the Akt phosphorylation protein level which was reversed by PI3K inhibitor ly294002. In addition, vaspin increased the phosphorylation levels of mTOR and p70S6K, which was inhibited by rapamycin. Meanwhile, we found that the NF-κB mRNA and protein levels were reduced after vaspin treatment, similar to the effect of NF-κB inhibitor TPCK. Furthermore, vaspin increased the glucose stimulated insulin secretion (GSIS) level, lowered blood glucose level and improved the glucose tolerance and insulin sensitivity of high fat diet fed rats. Hyperglycemic clamp test manifested that vaspin improved islet β cell function. Together, these findings provide a new understanding of the function of vaspin on pancreatic β cell and suggest that it may serve as a potential agent for the prevention and treatment of type 2 diabetes.

## Introduction

With the improvement of people's living standard and the change of lifestyle, the prevalence of obesity and obesity-induced diabetes have increased dramatically over the past several decades. According to the International Diabetes Federation (IDF) statistics, the number of patients with diabetes is about 415 million all over the world in 2015[1]. It is estimated that there will be 642 million people affected by diabetes in 2040, of which about 90% belong to type 2 diabetes. Type 2 diabetes mellitus has become one of the three major chronic noncommunicable diseases after cancer and cardiovascular disease, which seriously threaten human health[2].

It is well known that insulin resistance (IR) and islet β cell dysfunction are main pathophysiological features of type 2 diabetes. Islet β cells play a dual role in the regulation of blood glucose, they secrete insulin and accept the regulation of insulin simultaneously[3]. As the primary regulator of the insulin signaling pathway, tyrosine phosphorylation of IRS-2 can activate the phosphatidylinositol 3-kinase/protein kinase B (PI3K/Akt) signaling pathway. Then, Akt regulates many substrates activation and promotes cell growth and proliferation by activating the mTOR/p70S6K signaling pathway[4–7]. Therefore, any obstacles in PI3K/Akt insulin signaling pathway will lead to insulin resistance of islet β cells and result in the reduction of β cell function[8]. In addition, prolonged activation of mTOR can activate the p70S6K dependent negative feedback loop, leading to increased serine phosphorylation of IRS and down regulation of PI3K/Akt, which is involved in insulin resistance[9–14].

Inflammation is also known to be involved in the occurrence of type 2 diabetes. Inflammatory factors have been reported to accelerate the progress of insulin resistance. A number of recent studies have also shown that islet inflammation plays an important role in the pathogenesis of β cell failure in type 2 diabetic patients [15–18]. In addition, NF-κB is a key regulator in the activation and occurrence of chronic inflammatory response[19]. Activation of NF-κB has been implicated as a key event in the pathogenesis of diabetes and its associated complications[20]. Additionally, NF-κB is an intracellular target for hyperglycemia and hyperlipidemia [21], and the phosphorylation of the inhibitor IκB[22] is the major regulatory steps of NF-κB activation. IκB kinase (IKK) plays a crucial role in the phosphorylation of inhibitory κB (IκBs). At the same time, IKK is the serine kinase of insulin receptor and IRS-1, which can active the phosphorylation of IRS1-Ser307, and result in insulin resistance[23]. Studies have shown that inhibiting IKKβ activity or knocking out the gene can improve insulin resistance[24].

Vaspin was isolated from visceral white adipose tissues (WATs) of Otsuka Long-Evans Tokushima fatty (OLETF) rat, an animal model of abdominal obesity with type 2 diabetes[25]. Research has shown that vaspin possesses insulin sensitizing effect, can improve insulin sensitivity in obese mice induced by high-fat/high-glucose diet[26]. Meanwhile, vaspin has anti-inflammatory action. It can suppress proinflammatory cytokine mediated activation of NF-κB and the expression of downstream molecules, protecting vascular endothelial cell through inhibiting inflammation[27]. Thus, the present study was performed to investigate whether vaspin can act on IRS/PI3K/Akt insulin signaling pathway and NF-κB inflammatory signaling pathway to improve pancreatic β cell secretion function and insulin resistance, and reduce islet inflammation. The studies were performed in vivo using high fat diet fed rats and in vitro using the INS-1 cell models.

## Materials and methods

### Materials

Cell culture medium and fetal bovine serum were purchased from Gibco (Grand Island, NY, USA). Penicillin-streptomycin, HEPES, sodium pyruvate, β-mercaptoethanol, Enhanced Chemiluminescence (ECL) western blot detection system were purchased from Beyotime (Shanghai, China). Recombinant human vaspin was from Phoenix (San Francisco, CA, USA). Total RNA Extractor (Trizol) kit, the M-MLV RTase cDNA kit, RIPA lysate and the BCA protein assay kit were obtained from Boster (Wuhan, China). Antibodies against IRS-2, p-IRS-2 (Ser731), Akt, p-Akt (Thr308), mTOR, p-mTOR (Ser2448), p70S6K, p-p70S6K (Thr389), NF-κB P65 were purchased from Cell Signaling Technology (Beverly, MA, USA). PVDF membrane was obtained from Millipore (Germany). Rat insulin ELISA kit was from Westang (Shanghai, China). Cell Counting Kit-8 (CCK-8) was obtained from Boster (Wuhan, China).

### Cell culture

Rat insulinoma cells (INS-1) were purchased from the Institute of Basic Medical Sciences of the Chinese Academy of Medical Sciences (Beijing, China). All cells were cultured in RPMI1640 medium supplemented with 12% fetal bovine serum (FBS), penicillin-streptomycin (500 U/ml), 10mM HEPES, 0.11 g/L sodium pyruvate, and 50 μM β-mercaptoethanol in humidified 5% CO_2_ at 37°C.

### Cell treatment

For determining the appropriate concentration of vaspin, INS-1 cells were cultured and divided into five groups: (1) control group: treated with culture medium only; (2) PA group: treated with 0.5 mmol/L palmitic acid (PA); (3) PA + vaspin (80 ng/ml): treated with 0.5 mmol/L palmitic acid and 80 ng/ml vaspin; (4) PA + vaspin (160 ng/ml): treated with 0.5 mmol/L palmitic acid and 160 ng/ml vaspin; (5) PA + vaspin (320 ng/ml): treated with 0.5 mmol/L palmitic acid and 320 ng/ml vaspin.

In order to detect whether vaspin can improve insulin secretion function of INS- 1 cell through PI3K/Akt signaling pathway, cells were assigned into three groups: (1) PA group: treated with 0.5 mmol/L palmitic acid; (2) PA + vaspin group: treated with 0.5 mmol/L palmitic acid and 320 ng/ml vaspin; (3) PA + vaspin + ly294002 group: treated with 0.5 mmol/L palmitic acid and 320 ng/ml vaspin and 25 μmol/L ly294002 (PI3K inhibitor).

Based on the biological effects of mTOR/S6K1 signaling pathway, we further explored whether vaspin can improve cell proliferation of INS-1 cells through mTOR/S6K1 signaling pathway. Cells were assigned into five groups: (1) NC group: treated with serum-free medium; (2) PA group: treated with 0.5 mmol/L palmitic acid; (3) PA + vaspin group: treated with 0.5 mmol/L palmitic acid and 320 ng/ml vaspin; (4) PA + vaspin + Rapamycin group: treated with 0.5 mmol/L palmitic acid and 320 ng/ml vaspin and 25 nmol/L rapamycin; (5) PA + Rapamycin group: treated with 0.5 mmol/L palmitic acid and 25 nmol/L rapamycin (mTOR inhibitor, served as positive control).

In order to determine whether vaspin can improve insulin secretion function of INS-1 cell via NF-κB signaling pathway, cells were assigned into three groups: (1) PA group: treated with 0.5 mmol/L palmitic acid; (2) PA + vaspin group: treated with 0.5 mmol/L palmitic acid and 320 ng/ml vaspin; (3) PA + TPCK group: treated with 0.5 mmol/L palmitic acid and 20 μmol/L TPCK (NF-κB inhibitor, serves as positive control).

### Animals

Thirty male Sprague-Dawley rats (200–220 g) were purchased from Beijing Vital River Laboratory Animal Technology Co., Ltd (Beijing, China). The rats were housed in the Laboratory Animal Center of Shanxi Medical University, which is a specific pathogen free facility. All animals were maintained at 22–26°C temperature, 40–60% humidity and a 12-h light/12-h dark cycle in the animal facility with free access to food and water.

### Animals treatment

As a first step, the rats were randomly assigned into normal diet group (NC group, n = 10) and high-fat-diet group (HFD group, n = 20) after one week of adaptive feeding. The normal diet contained 57% carbohydrate, 18% protein, and 25% fat; the high-fat diet contained 37% carbohydrate, 13% protein and 50% fat. After 16 weeks of dietary manipulation, rats were randomly divided into high fat diet group (HF group, n = 10) and vaspin treatment group (HF + vaspin group, n = 10). Rats in the vaspin group were treated with 320 ng/ml (3ml/kg) vaspin intraperitoneally once daily for 4 weeks. The NC group and HF group were given the same volume of normal saline solution (as vehicle control) for the same duration as vaspin. The body weight was recorded weekly. Fasting blood glucose (FBG) was measured biweekly from tail vein using a Freestyle Blood Glucose Meter (Johnson & Johnson, New Jersey, USA).

All animal procedures were approved by the Institutional Animal Care and Use Committee of Shanxi Medical University and conformed with Guide for the Care and Use of Laboratory Animals 8th Edition 2011[28]. All efforts were made to minimize suffering during animal experiments. Hyperglycemic clamp test was performed under sodium pentobarbital anesthesia. Upon termination of each experiment, animals were euthanized by intraperitoneal injection of sodium pentobarbital (200 mg/kg body weight).

### Oral glucose tolerance test (OGTT)

The oral glucose tolerance test (OGTT) was performed in overnight-fasted rats at the end of intervention in all rats. Each experimental animal received a single dose of 1.5 g of 50% glucose solution/kg body weight via gavage in the morning. Blood samples were obtained from the tail vein and glucose values were measured by the glucose meter before glucose loading (t = 0) and 30, 60, and 120 min after glucose administration. Feed were resumed on completion of the test. The area under the curve for glucose (AUC glucose) was used to assess the pancreatic β cell function.

### Insulin tolerance test (ITT)

After one day recovery, animals were overnight-fasted and intraperitoneally injected with Recombinant Human Insulin (1 IU/kg body weight; Humulin R, Eli Lilly and Company, Indianapolis, IN, USA). Blood samples were obtained from the tail vein. The glucose values were measured with the glucose meter before loading (t = 0) and 30, 60, 120 min after insulin administration. The test was performed in the morning, and the feed were resumed on completion of the test.

### Hyperglycemic clamp test

Hyperglycemic clamp test was performed on consecutive days in all animals. Rats were fasted overnight and anesthetized with sodium pentobarbital (50 mg/kg body weight) via intraperitoneal injection. A catheter was placed in the right jugular vein for 50% glucose infusion. Blood samples were taken via tail vein for determination of insulin concentrations. The blood glucose was measured at time 0, immediately after the initial injection of glucose, and continuously monitored at 1, 5, 10, 15 min and every five minutes thereafter until it maintained at (14 ± 0.5) mmol/L for 5 consecutive time points by adjusting the infusion rate of the 50% glucose solution. In addition, approximately 100 μl of blood was collected at 0, 1, 5, 10, 15 min and any time point in the steady state. Plasma was obtained by centrifuging blood sample (4500 rpm, 15 min, 4°C) and stored at -20°C. The reaction of islet β cells to glucose and the islet secretion capacity were assessed according to the GIR (glucose infusion rate). GIR(mg/kg·min) = Rate (μl/min) × glucose concentration (g/ml) ÷ 1000 ÷ B.W.(g). The insulin level was used to evaluate bidirectional regulating effects of islet β cells.

### Real-time PCR

Total RNA was extracted from INS-1 cells using the Total RNA Extractor (Trizol) kit. cDNA of each RNA sample was reverse transcribed with the M-MLV RTase cDNA kit according to the manufacturer’s instruction. Real-time PCR was performed using SYBR Premix EX Tap 2x kit in CFX 96 PT-PCR system. The reaction conditions were: 95°C for 30 s, 95°C for 5 s, 60°C for 30 s, followed by 40 cycles and then 72°C for 10 min. Primer sequences were as follow: IRS-2: F: 5′-CTACCCACTGAGCCCAAGAG-3′, R: 5′-CCAGGGATGAAGCAGGACTA-3′; Akt: F: 5'-GAAGACCCAAAGACCAAGATGC-3′, R: 5'-TCTGACAACAAAGCAGGAGGTG-3′; NF-κB p65: F: 5′-CATGCCAACGCCCTCTTCGA-3′, R: 5′-TGTCCCCGTTCTCATCCTGCAC-3′; β-actin: F: 5′-CTAGAAGCATTTGCGGTGGA-3′, R: 5′-GAAATCGTG CGTGACATTAAG-3′. β-actin was used as an internal control for normalization of the gene expression data using the real-time quantitative PCR and the 2 (-Delta Delta C (T)) Method[29].

### Western blot analysis

To extract protein, cells were lysed on ice for 10 min in RIPA buffer and then each emulsion was centrifuged (14,000 rpm, 4°C for 15 min) and supernatant was collected. The protein concentrations were quantified by BCA protein assay kit. Equal amounts of proteins were separated by SDS/PAGE (10% gels) and then transferred to a PVDF membrane. After blocking with 5% nonfat dry milk, the membranes were incubated with primary antibody overnight at 4°C, then washed with TBST and incubated with horseradish peroxidase (HRP)-conjugated secondary antibody for additional 2 h at room temperature. The blots were visualized by enhanced chemiluminescence (ECL) western blot detection system. The signal intensity was quantified using Image J software.

### Glucose-stimulated insulin secretion

INS-1 cells of same amount (2.5×10^5^ cell/well) were seeded in 24-well culture dishes and treated with glucose (16.7 mmol/L) for 1 h. The concentration of insulin from the supernatant was determined using ELISA kit.

### Cell proliferation assay

The proliferation activity of INS-1 cells was measured using the Cell Counting Kit-8 (CCK-8). INS-1 cells were assigned into five groups: NC group, PA group, PA + vaspin group, PA + vaspin + Rapamycin group, PA + Rapamycin group. In each experiment, a blank and a negative control group were included. After the intervention, a volume of 10 μL of CCK-8 solution was added to each well 4 hours before the completion of incubation. The viability of the cells was determined using a spectrophotometer plate reader at an absorbance of 450 nm.

### Statistical analysis

Data analysis were performed with SPSS 20.0 (SPSS Inc., Chicago, IL, USA). The results were presented as the mean ± SD. If the data distribute normally and the variance is homogeneous, differences between two groups were assessed with Student's unpaired and two-tailed t tests. One-way analysis of variance followed by least significant difference (LSD) t-test was used to calculate differences among the various study groups. If the data does not obey normal distribution or the variance is not uniform, non-parametric test (kruskal wallis test) were used to analyze differences between groups. *P* < 0.05 was considered statistically significant.

https://dx.doi.org/10.17504/protocols.io.kjicuke.[PROTOCOL DOI]

## Results

### Vaspin lowered blood glucose level and improved the glucose tolerance and insulin sensitivity in high fat diet fed rats

**Fig 1:**
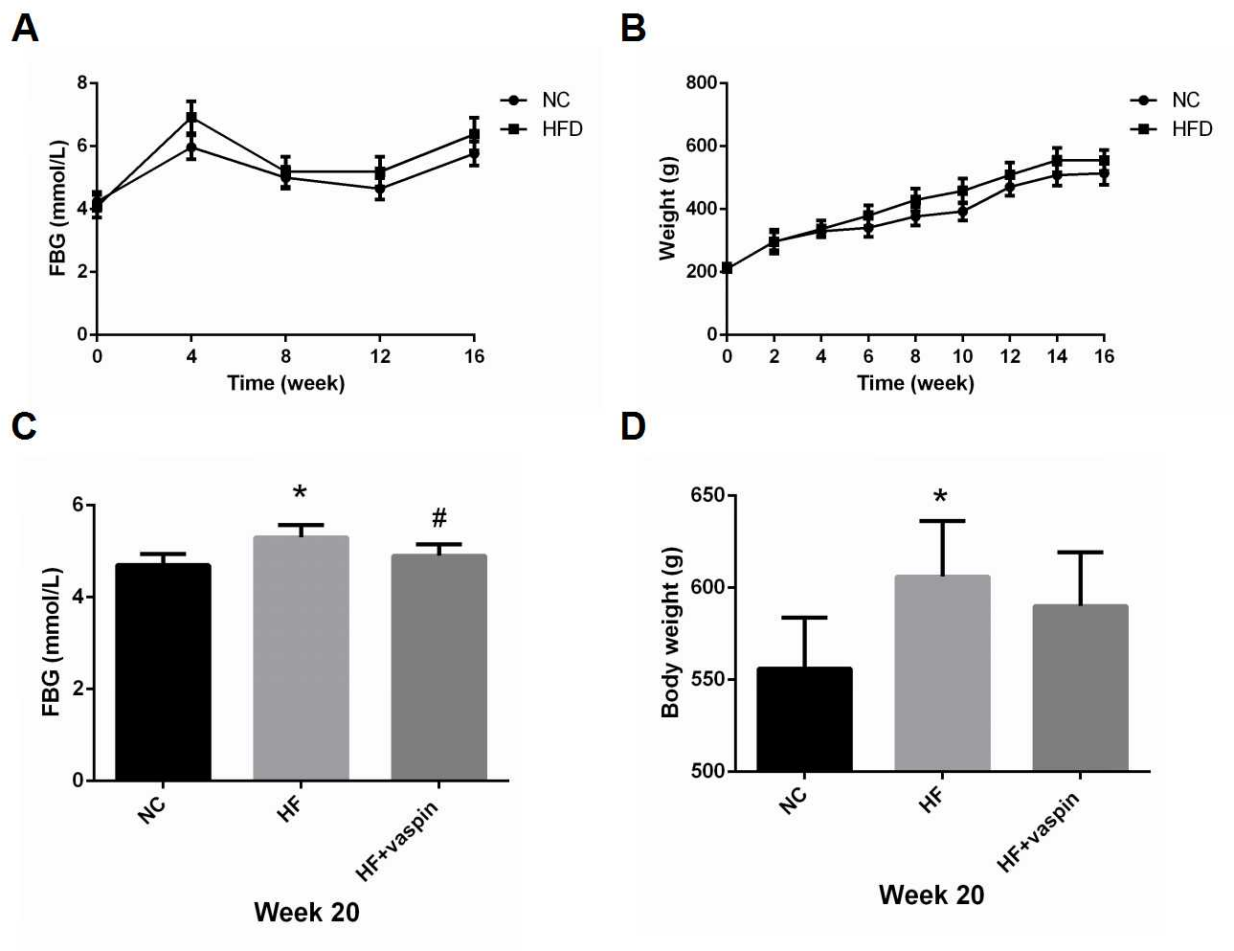
Effects of vaspin on blood glucose level and body weight. The rats were randomly assigned into NC group (n = 10) and HFD group (n = 20). The body weight of the rats was measured weekly, fasting blood glucose (FBG) of the rats was measured biweekly. (A-B) The trends of blood glucose and body weight in rats fed with normal diet and high fat diet before intervention. The results were compared and analyzed with Student's unpaired and two-tailed t tests. (C-D) After 16 weeks of dietary manipulation, high-fat-diet rats were randomly divided into HF group (n = 10) and HF plus vaspin group (n = 10). At the end of intervention with vaspin (20 ng/ml, 3ml/kg) intraperitoneally once daily for 4 week, blood glucose level and body weight were compared between the groups. One-way analysis of variance followed by least significant difference (LSD) t-test was used to calculate differences among the various study groups. Data were shown as mean ± SD. * *P < 0*.*05* as compared with NC group; # *P < 0*.*05* as compared with HF group.

**Fig 2:**
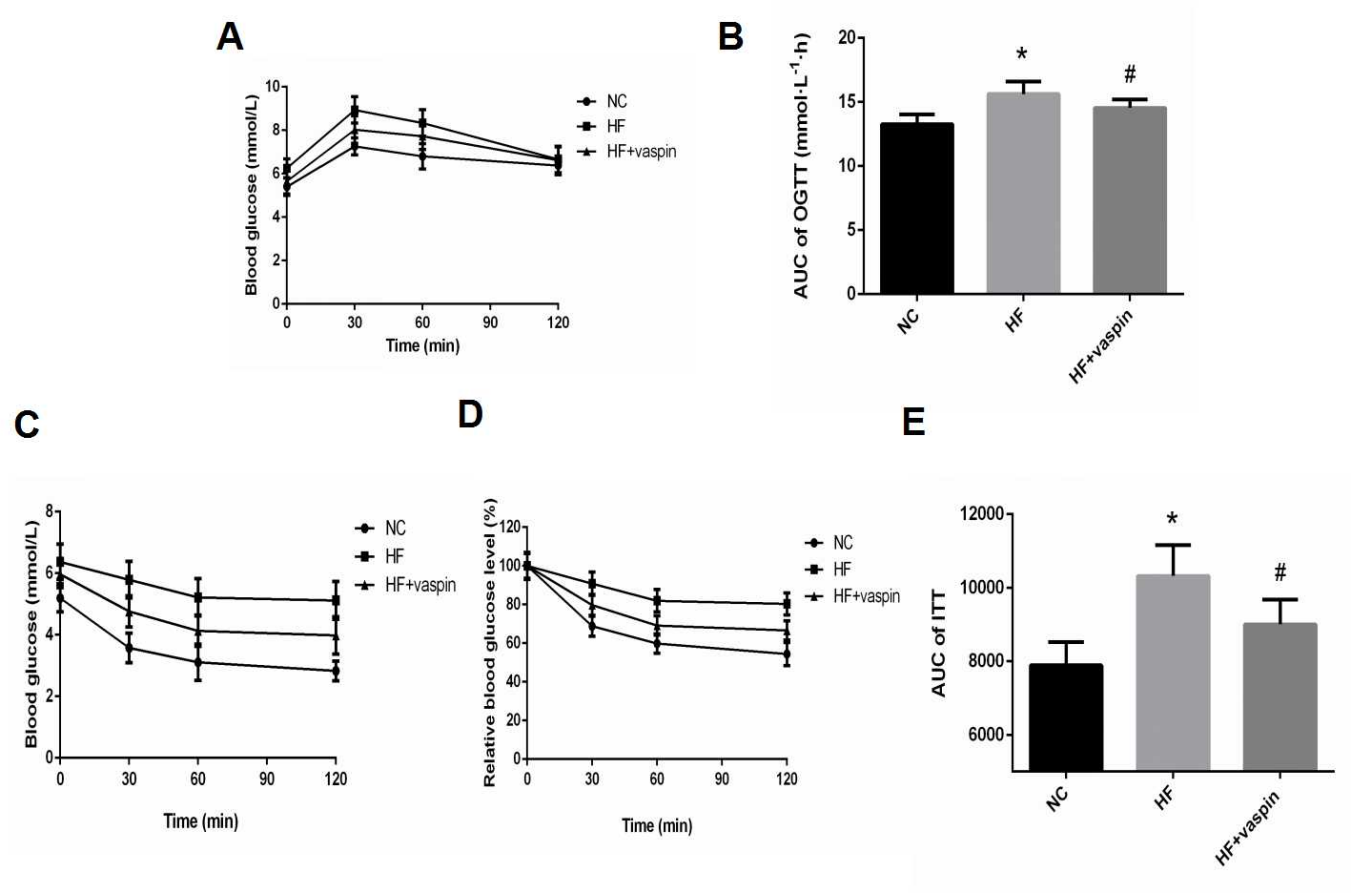
Effects of vaspin on OGTT and ITT in different group rats, n = 10/group. At the end of intervention with vaspin, the oral glucose tolerance test (OGTT) and insulin tolerance test (ITT) were performed in overnight-fasted rats. (A) oral glucose tolerance test (OGTT). (B) area under curve of OGTT. (C) insulin tolerance test (ITT). (D) ITT show as a percentage. (E) area under curve of ITT. Data were shown as mean ± SD. The results were analyzed with One-way analysis of variance followed by LSD test. * *P < 0*.*05* as compared with NC group; # *P < 0*.*05* as compared with HF group.

Effects of vaspin were examined in vivo using rats fed with high fat diet. During the 16 weeks’ high-fat-diet feeding, blood glucose levels fluctuated in both control and HFD fed rats without significant difference between the two groups during the whole period (Fig 1A). Body weights of the rats were steadily increased in both groups after 16 weeks but not significantly different at any of the time points examined (Fig 1B). Vaspin treatment significantly reduced blood glucose level of HF rats (Fig 1C), while having no significant effect on body weight (Fig 1D). Meanwhile we found that vaspin improved glucose tolerance and insulin sensitivity as evidenced by a significantly lower plasma glucose level and decreased area under OGTT and ITT curve in the HF rats treated with vaspin in comparison with those untreated ones (Fig 2).

### Vaspin improved islet β cell function in high fat diet fed rats

**Fig 3:**
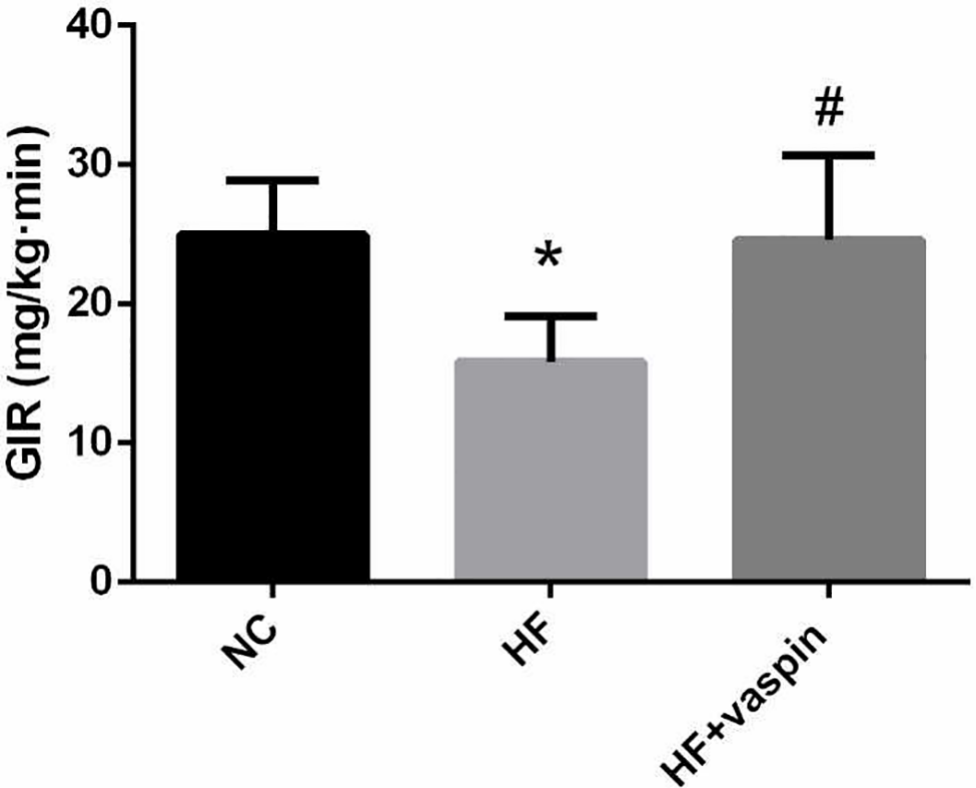
Results of hyperglycemia clamps test in different group rats, n = 10/group. At the end of intervention with vaspin, hyperglycemic clamp test was performed in overnight-fasted rats. The reaction of islet β cells to glucose and the islet secretion capacity were assessed according to the GIR. Data were shown as mean ± SD. The data obey normal distribution, but the variance was not uniform, thus Kruskal wallis test was used to analyze the difference in different groups rats. * *P < 0*.*05* compared with NC group; # *P < 0*.*05* compared with HF group.

Hyperglycemic clamp test showed that vaspin significantly improved islet β cell function of HF rats. GIR was used to assess the reaction of islet β cells to glucose and the secretion capacity of islet β cells. As expected, the GIR in the HF rats was significantly lower than that of control group, which suggested that vaspin could improve islet β cell function (Fig 3).

### Effects of vaspin on IRS-2 mRNA and protein levels in INS-1 cells

**Fig 4:**
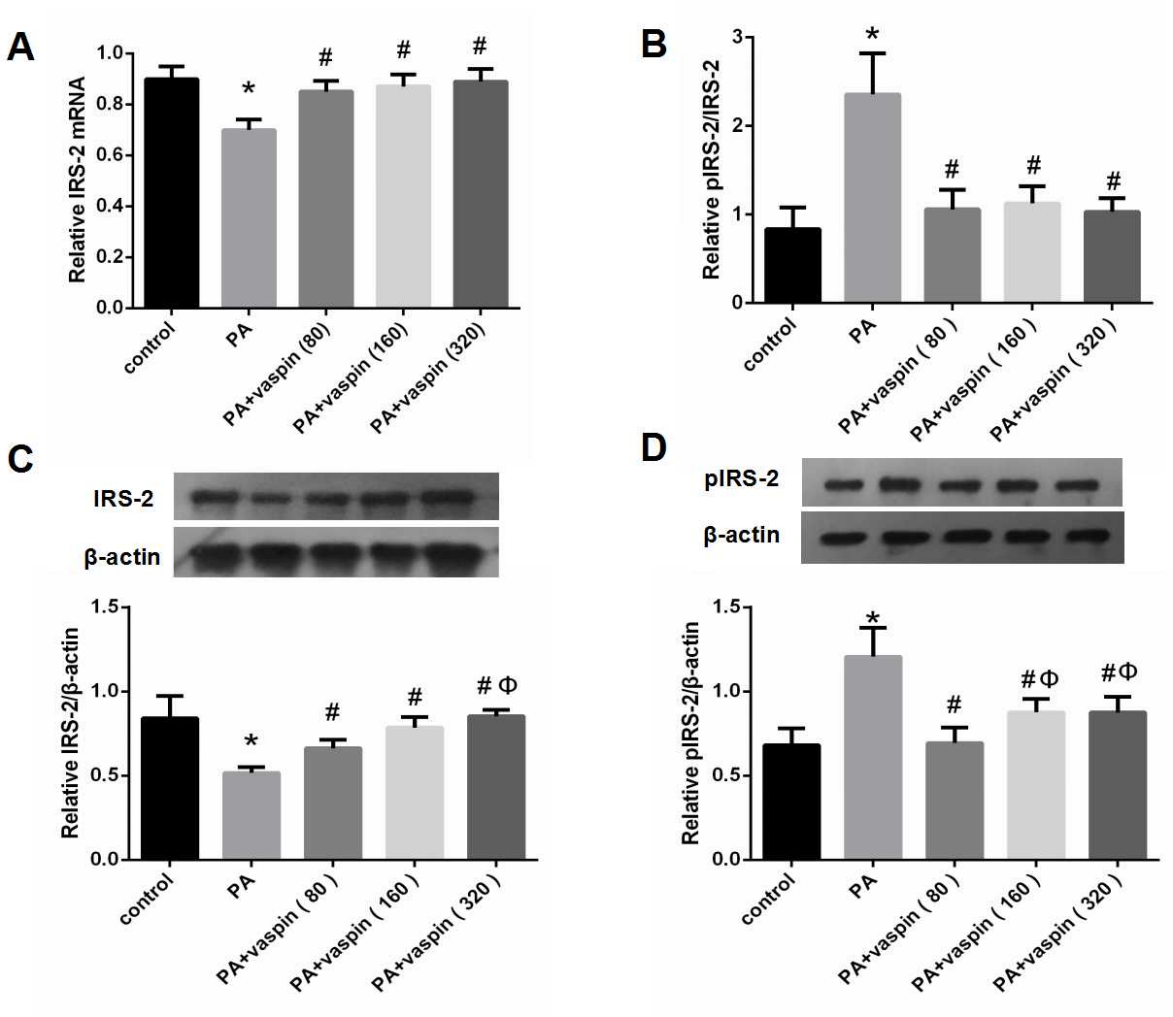
Effects of vaspin on IRS-2 mRNA and protein levels in INS-1 cells. INS-1 cells were treated with 0 (control), 80, 160, 320 ng/ml vaspin for 24 h. (A) Effect of vaspin on IRS-2 mRNA in INS-1 cells. The level of IRS-2 mRNA was determined by Real-time PCR. (B-D) Effects of vaspin on the protein levels of total IRS-2, pIRS-2 and pIRS-2/IRS-2 ratio in INS-1 cells. Cell lysates were subjected to western blotting and incubated with antibodies against IRS-2 and pIRS-2. Data were shown as mean ± SD of at least three independent experiments. Differences among groups were compared with one-way analysis of variance followed by LSD test. * *P* < 0.05 compared with control group; # *P* < 0.05 compared with PA group; Φ *P* < 0.05 compared with PA plus 80 ng/ml vaspin group.

Compared with control group, both the mRNA and total protein levels of IRS-2 in the PA group were significantly decreased following PA treatment. In contrast, the serine phosphorylation level of IRS-2 was increased, resulting in a raised pIRS-2 and IRS-2 ratio in PA treated cells (Fig 4A–4D). This indicates that PA could suppress the expression of IRS-2 mRNA and protein, while promoting the serine phosphorylation of IRS-2 protein.

Meanwhile, INS-1 cells were treated with various concentrations of vaspin (80, 160 and 320 ng/ml) for 24 h, the levels of IRS-2 mRNA and total protein in PA plus vaspin intervention groups were significantly increased compared with PA intervention group (Fig 4A and 4C). However, the serine phosphorylation level of IRS-2 protein in PA plus vaspin group was significantly reduced when compared to PA group (Fig 4D), which resulted in the reversion of the pIRS-2/IRS-2 ratio (Fig 4B). This demonstrated that vaspin could enhance the expression of IRS-2 mRNA and total protein, while inhibited the serine phosphorylation level of IRS-2 protein induced by palmitic acid.

### Effects of vaspin on PI3K/Akt signaling pathway in INS-1 cells

**Fig 5:**
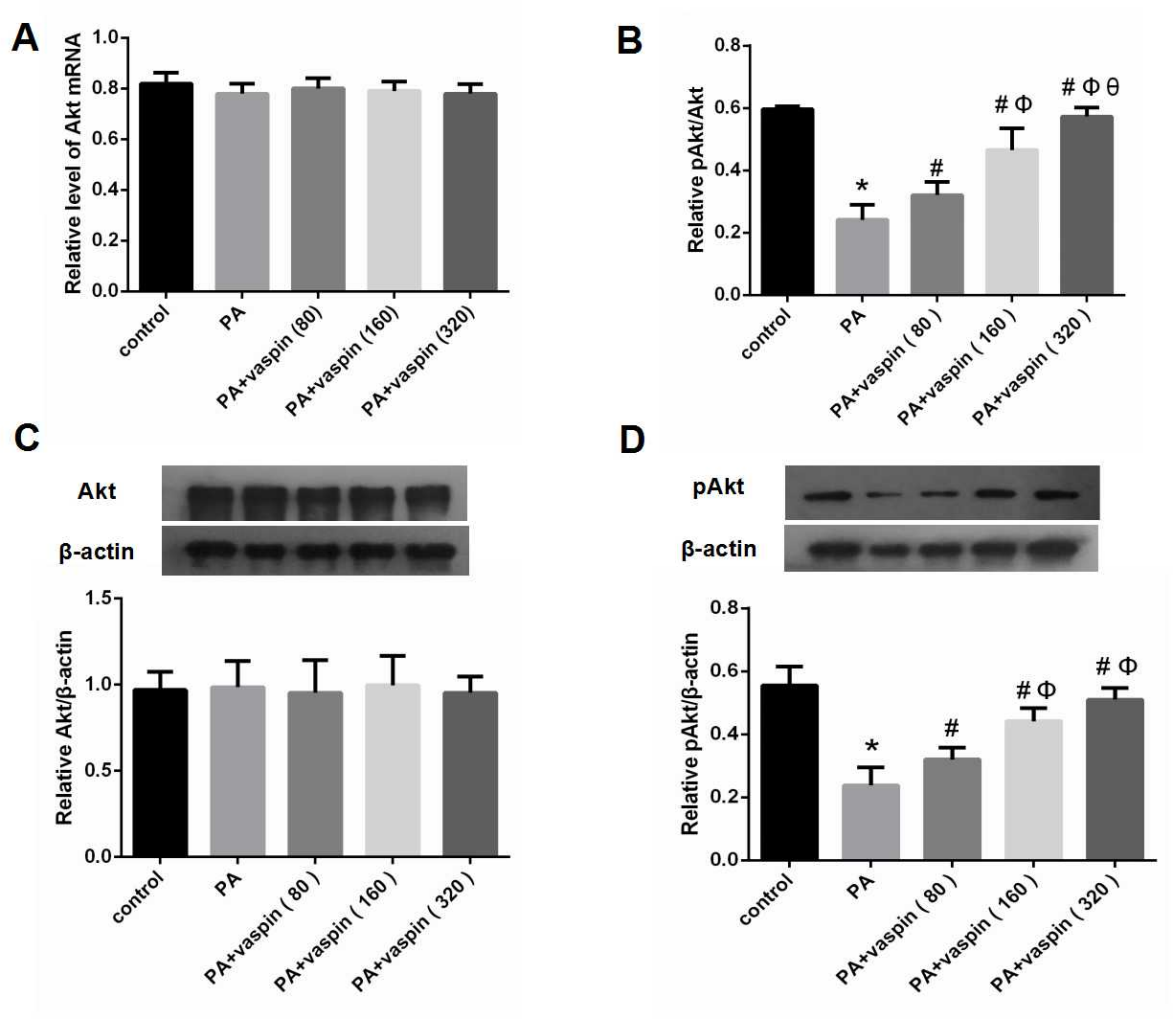
Effects of vaspin on PI3K/Akt signaling pathway in INS-1 cells. INS-1 cells were treated with 0 (control), 80, 160, 320 ng/ml vaspin for 24h. (A) Effect of vaspin on Akt mRNA in INS-1 cells. The levels of Akt mRNA were determined by Real-time PCR. (B-D) Effects of vaspin on the protein levels of total Akt, pAkt and pAkt/Akt in INS-1 cells. Cell lysates were subjected for western blotting and incubated with antibodies against Akt and pAkt. Data were shown as mean ± SD of at least three independent experiments. One way analysis of variance followed by LSD test was used compare differences between experimental groups and control group. * *P* < 0.05 compared with control group; # *P* < 0.05 compared with PA group; Φ *P* < 0.05 compared with PA plus 80 ng/ml vaspin group; θ *P* < 0.05 compared with PA plus 160 ng/ml vaspin group.

The levels of Akt mRNA and protein were not significantly different between the PA group and control group (Fig 5A and 5C), however the phosphorylation level of Akt was decreased significantly in PA group (Fig 5D). Higher phosphorylation level of Akt protein was detected in PA plus vaspin intervention groups, as compared with PA group (Fig 5D). Accordingly, the pAkt to Akt ratio was significantly increased in PA plus vaspin groups when compared to PA group (Fig 5B). As noted, the most remarkable increase was seen in cells treated with 320 ng/ml vaspin, suggesting that co-treatment with vaspin rescued the reduction of phosphorylation level of Akt protein induced by palmitic acid (Fig 5).

### Vaspin promoted the phosphorylation of Akt through PI3K signaling pathway

**Fig 6:**
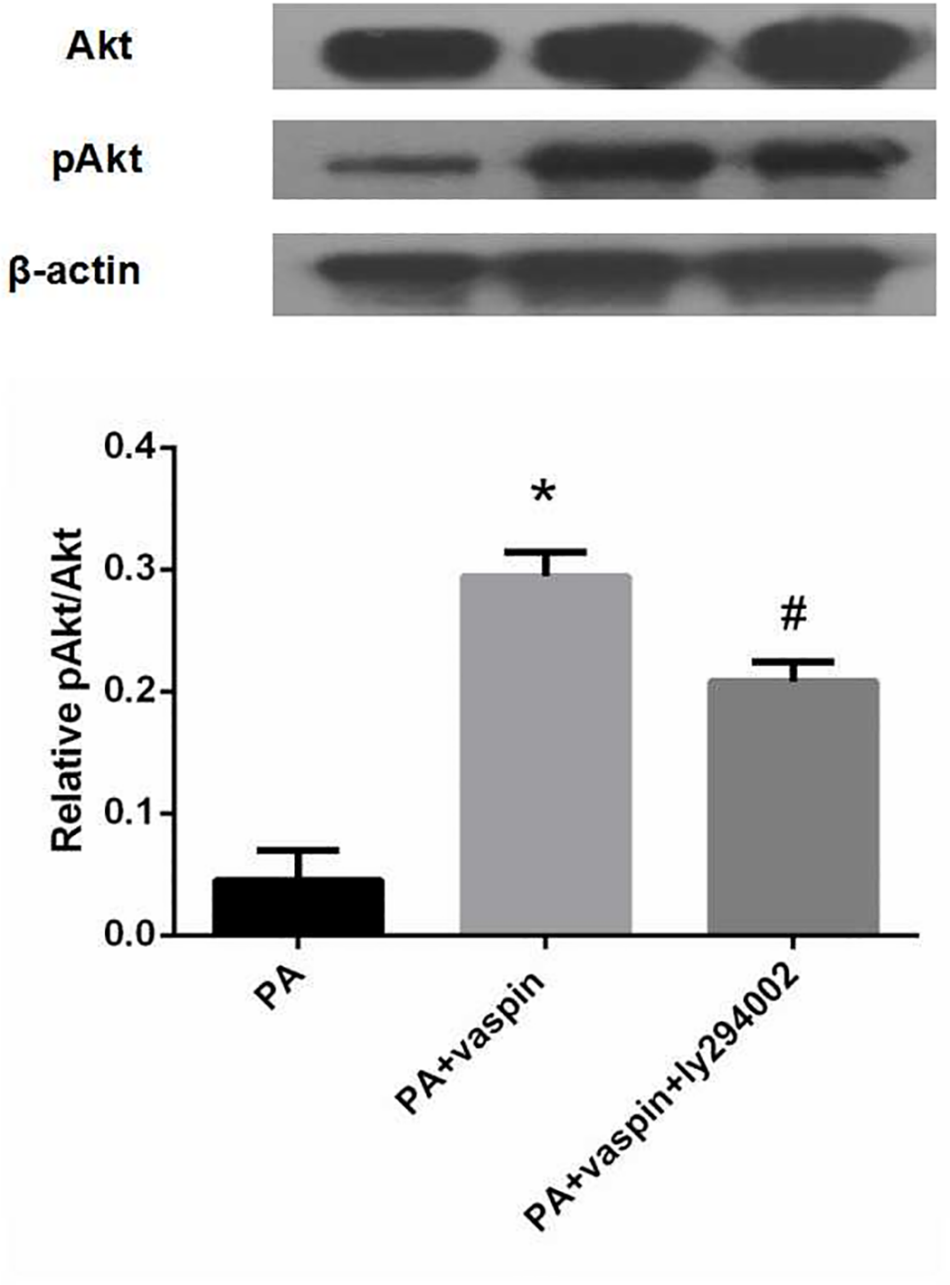
Vaspin promotes the phosphorylation of Akt by activiting PI3K. INS-1 cells of same amount (2.5×10^5^ cell/well) were treated with serum-free medium for 24 h. The medium was then replaced by fresh serum-free medium containing 0.5 mmol/L palmitic acid alone or 0.5 mmol/L palmitic acid plus vaspin (320 ng/ml) in the absence or presence of 25 μmol/L ly294002 for 24 h. The protein levels of Akt, pAkt were determined by western blot analysis. Data were shown as mean ± SD of at least three independent experiments. One way analysis of variance followed by LSD test was used to calculate differences among the various study groups. * *P* < 0.05 compared with PA group; # *P* < 0.05 compared with PA plus vaspin group.

The phosphorylation of Akt can be regulated by PI3K, as well as other second messengers such as Ca^2+^ and cAMP[30]. Thus, we also examined whether vaspin could promote the phosphorylation of Akt through PI3K signaling pathway. We found that addition of PI3K inhibitor ly294002 revered the elevated phosphorylation level of Akt following vaspin treatment, suggesting that vaspin promoted the phosphorylation of Akt through PI3K signaling pathway (Fig 6).

### PI3K/Akt signaling pathway mediated the effects of vaspin on insulin secretion

**Fig 7:**
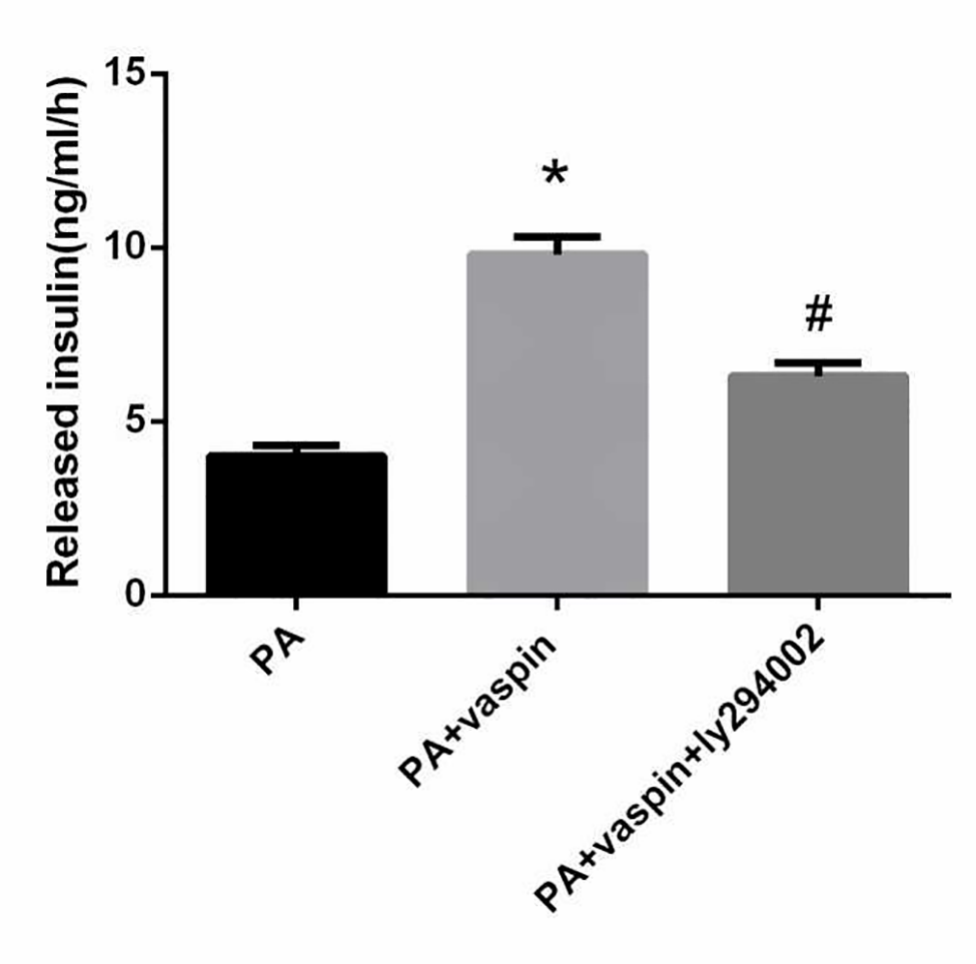
Effect of vaspin on the function of INS-1 was mediated by PI3K/Akt signaling pathway. INS-1 cells were seeded in 24-well culture dishes and treated with serum-free medium for 24 h. The medium was then replaced by fresh serum-free medium containing 0.5 mmol/L palmitic acid alone or 0.5 mmol/L palmitic acid plus vaspin (320 ng/ml) in the absence or presence of 25 μmol/L ly294002 for 24 h. After intervention, INS-1 cells were treated with glucose (16.7 mmol/L) for 1 h after sugar-free cultured 1 h. Data were shown as mean ± SD of at least three independent experiments. Differences among groups were compared with one way analysis of variance followed by LSD test. * *P* < 0.05 compared with PA group; # *P* < 0.05 compared with PA + vaspin group.

As shown in Fig 7, vaspin intervention increased insulin secretion level of INS-1 pre-stimulated with glucose compared with PA group. Nevertheless, vaspin co-treatment with PI3K inhibitor ly294002 appeared to reverse the vaspin induced insulin stimulating effect in the INS-1 cells. This confirmed that vaspin can protect the secretion function of INS-1 cell via PI3K/Akt insulin signaling pathway (Fig 7).

### Vaspin promoted the phosphorylation of mTOR and p70S6K in vitro

**Fig 8:**
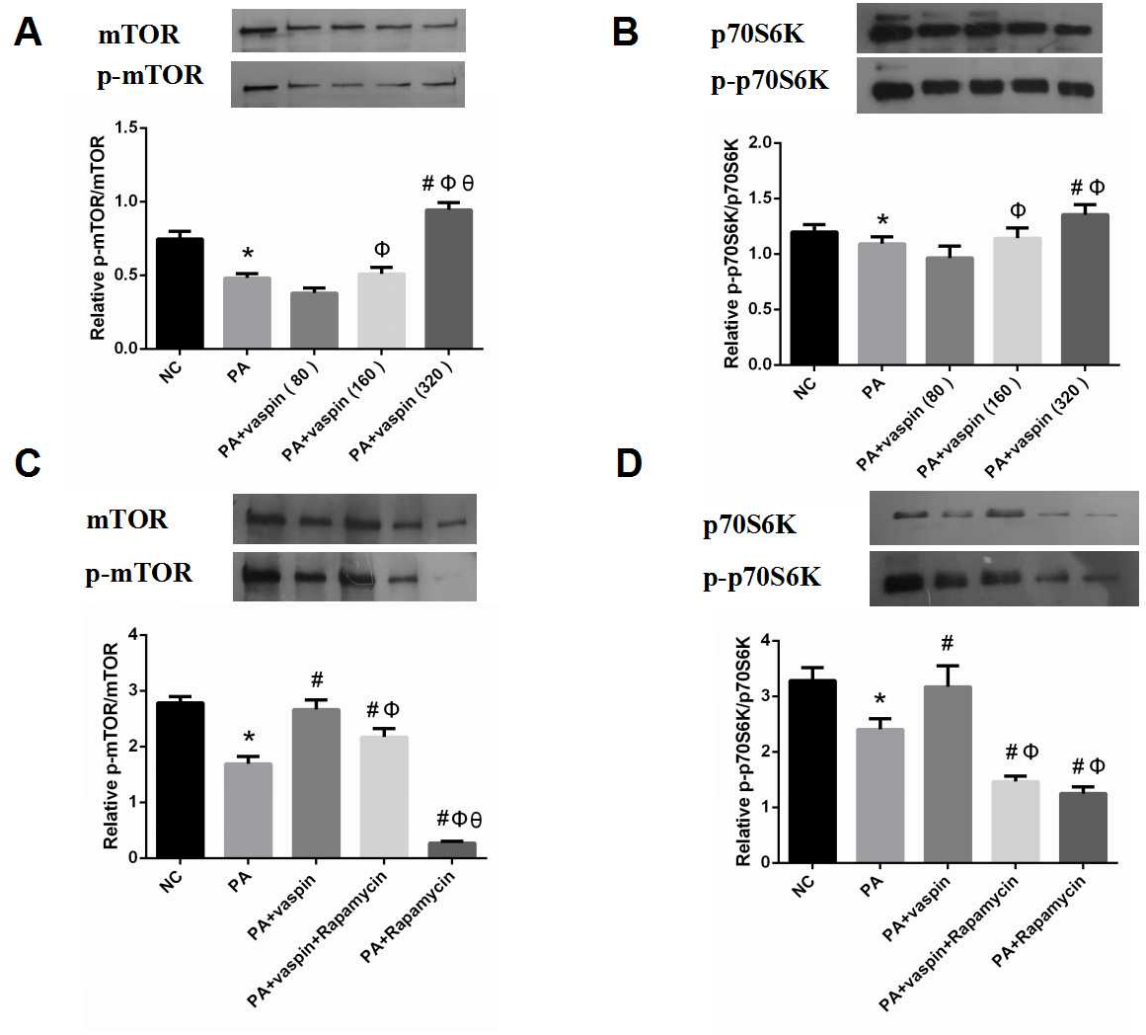
Effect of vaspin on mTOR/p70S6K signaling pathway in INS-1 cells. (A-B) INS-1 cells were treated with 0 (control), 80, 160, 320 ng/ml vaspin for 24 h. Cell lysates were subjected to western blotting and incubated with antibodies against mTOR, p-mTOR, p70S6K, p-p70S6K. (C-D) INS-1 cells of same amount (2.5×10^5^ cell/well) were incubated in serum-free medium for 24 h. The medium was then replaced by fresh serum-free medium in the absence or presence of 0.5 mmol/L palmitic acid alone for 24 h. Then, a final concentration of 25 nmol/L rapamycin or equivalent volumes of DMSO (vehicle) added to medium over 60 min. Finally, the cells were incubated with or without vaspin for 24 h. The protein levels of mTOR, p-mTOR, p70S6K, p-p70S6K were determined by western blotting analysis. Data were shown as mean ± SD of at least three independent experiments. The results were analyzed with One-way analysis of variance followed by LSD test. * *P* < 0.05 compared with NC group; # *P* < 0.05 compared with PA group; Φ *P* < 0.05 compared with PA plus vaspin group; θ *P* < 0.05 compared with PA +vaspin +Rapamycin group.

It is well known that the mTOR/p70S6K signaling pathway is involved in the pathogenesis of insulin resistance. mTOR is a positive regulator of protein translation, and phosphorylation of S6K is considered a marker for mTOR activation. As shown in Fig 8A and 8B, PA significantly inhibited the phosphorylation of mTOR and p70S6K in INS-1 cells, however, vaspin can reverse this effect. In addition, the mTOR inhibitor rapamycin was used to further verify whether vaspin promoted the phosphorylation of mTOR and p70S6K through mTOR pathway. Obviously, addition of mTOR inhibitor rapamycin reversed the elevated phosphorylation level of mTOR and p70S6K following vaspin treatment, suggesting that vaspin promoted the phosphorylation of p70S6K through mTOR signaling pathway (Fig 8C and 8D). This study suggests that vaspin can indeed act on the mTOR/p70S6K signaling pathway to improve insulin resistance.

### Vaspin could promote cell growth and proliferation through the mTOR/p70S6K signaling pathway in INS-1 cells

**Fig 9:**
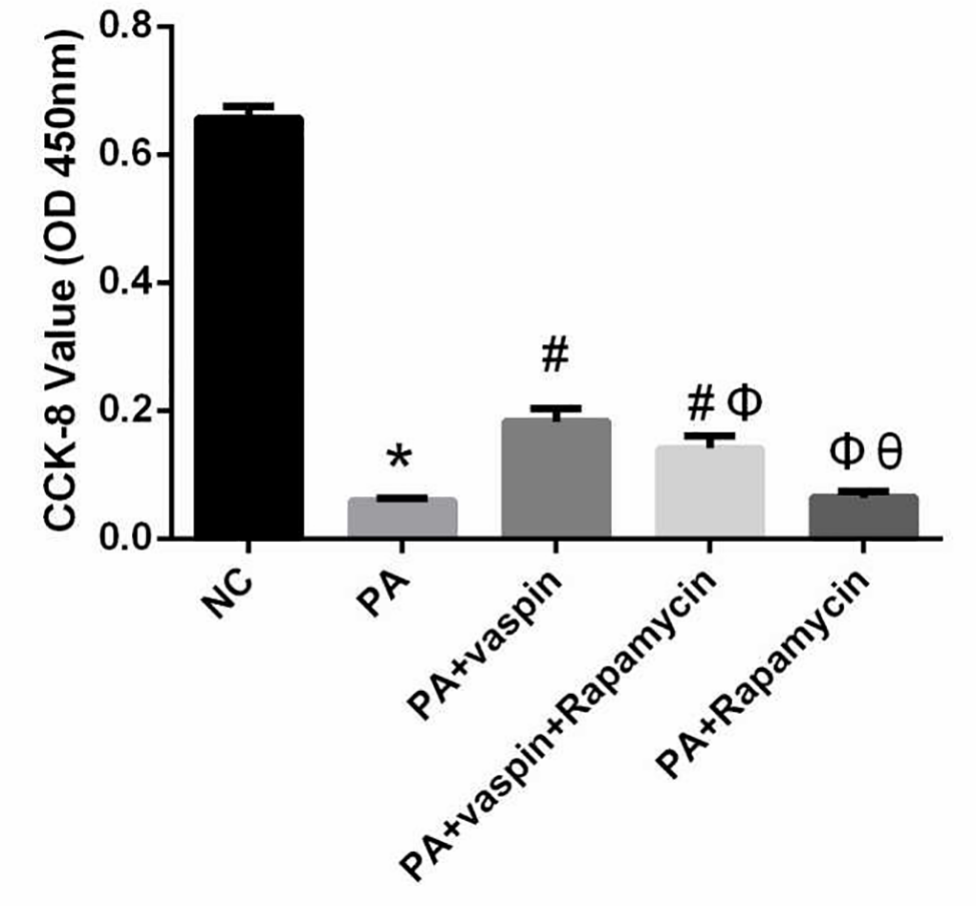
Effect of vaspin on INS-1 cell proliferation in vitro. INS-1 cells were assigned into five groups: NC group, PA group, PA + vaspin group, PA + vaspin + Rapamycin group, PA + Rapamycin group. The effect of vaspin on INS-1 cell proliferation was determined by CCK-8 assays. Data were shown as mean ± SD of at least three independent experiments. Groups comparison using one-way analysis of variance followed by LSD test. * *P* < 0.05 compared with NC group; # *P* < 0.05 compared with PA group; Φ *P* < 0.05 compared with PA plus vaspin group; θ *P* < 0.05 compared with PA +vaspin +Rapamycin group.

mTOR/p70S6K, as one of the downstream substrate of Akt, can promote cell growth and proliferation after activation. We used CCK-8 assays to determine whether vaspin could promote the proliferation of INS-1 cells and found that OD values of INS-1 cells treated with palmitic acid were lower than those of cells in NC group. When INS-1 cells were treated with 320 ng/ml vaspin, the OD values were higher than PA treated cells. It is noteworthy that rapamycin can inhibit the proliferation of INS-1 cells induced by vaspin (Fig 9).

### Effects of vaspin on NF-κB signaling pathway

**Fig 10:**
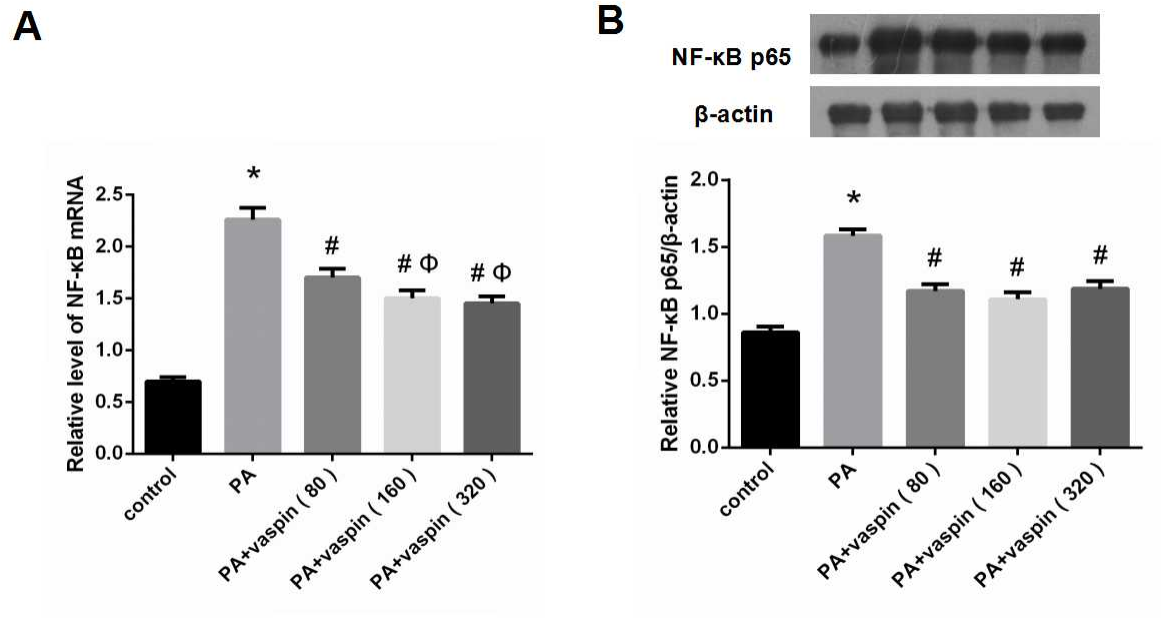
The effect of vaspin on NF-κB inflammation signaling pathway in INS-1 cells. INS-1 cells of same amount (2.5×10^5^ cell/well) in 24 well dishes were treated with 0 (control), 80, 160, 320 ng/ml vaspin for 24h. (A) Effect of vaspin on mRNA of NF-κB in INS-1 cells. The levels of NF-κB mRNA were determined by Real-time PCR. (B) Effects of vaspin on the protein levels of NF-κB p65 in INS-1 cells. Cell lysates were subjected to western blotting and incubated with antibodies against NF-κB p65. Data were shown as mean ± SD of at least three independent experiments. Multiple samples mean comparison was done using to one-way analysis of variance followed by LSD test. * *P* < 0.05 compared with control group; # *P* < 0.05 compared with PA group; Φ *P* < 0.05 compared with PA plus 80 ng/ml vaspin group; θ *P* < 0.05 compared with PA plus 160 ng/ml vaspin group.

**Fig 11:**
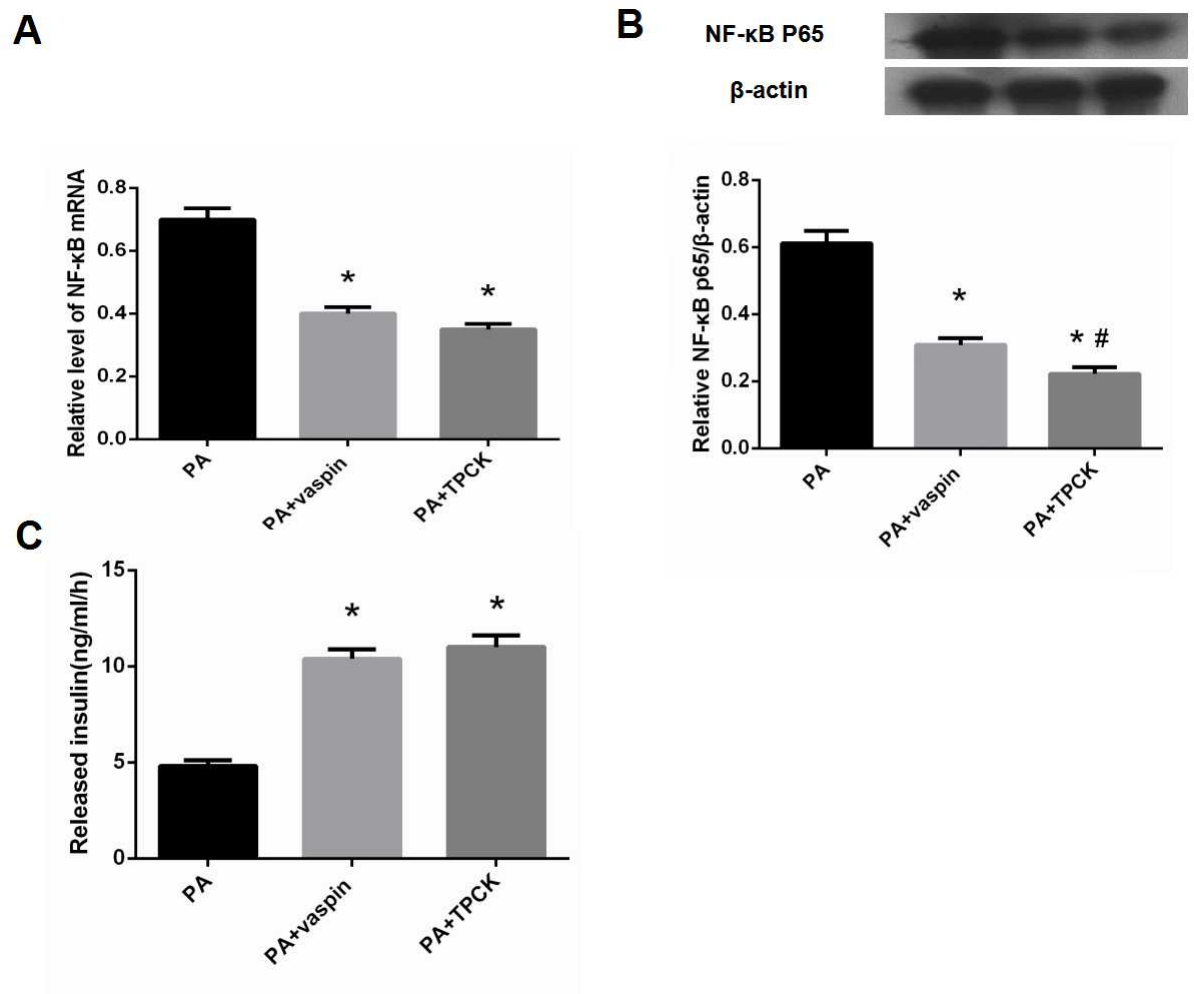
Effect of vaspin on INS-1 cell function was mediated by inhibiting NF-κB inflammation signaling pathway. INS-1 cells were seeded in 24-well culture dishes and treated with PA, PA + vaspin (320 ng/ml) and PA + TPCK (NF-κB inhibitor) 20 μmol/L for 24 h. (A) The level of NF-κB mRNA was determined by Real-time PCR. (B) The protein level of NF-κB p65 was determined by western blot analysis. (C) After intervention, INS-1 cells were treated with glucose (16.7 mmol/L) for 1 h following cultured 1 h in sugar-free medium. Data were shown as mean ± SD of at least three independent experiments. One-way analysis of variance followed by LSD test was used to calculate differences among the various study groups. * *P* < 0.05 compared with PA group; # *P* < 0.05 compared with PA + vaspin group.

In addition to the PI3K/Akt insulin signaling pathway, we also examined the effect of different levels of vaspin on the NF-κB signaling pathway. Compared with control group, NF-κB mRNA and p65 protein were significantly up-regulated in PA treated INS-1 cells (Fig 10A and 10B). However, they were clearly lower in cells treated with PA plus vaspin group than those in PA group (Fig 10A and 10B). Beyond that, the levels of NF-κB mRNA and p65 protein in PA plus TPCK group were significantly decreased as compared with PA group, which could also be seen in PA plus vaspin group (Fig 11A and 11B). This observation indicated that vaspin could block the activation of NF-κB signaling pathway induced by PA.

### Effect of vaspin on insulin secretion function of INS-1 cells was mediated by suppressing NF-κB signal pathway

The level of insulin secreted from INS-1 cells stimulated with glucose was increased in PA plus vaspin group compared with PA group (Fig 11C). Co-treatment of INS-1 cells with PA and TPCK (NF-κB inhibitor) also increased the insulin secretion compared with the PA group (Fig 11C). It suggested that vaspin could promote the secretion function of INS-1 cells by inhibition of NF-κB signaling pathway.

## Discussion

Insulin resistance is the main characteristic of type 2 diabetes mellitus. Study showed that insulin receptor signal transduction pathway components such as insulin receptor (IR), IRS, PI3K and its downstream protein kinase B (PKB) are also present in islet β cells in addition to the peripheral target tissues[8]. This suggests that pancreatic β cells can not only secrete insulin, may also be a target of insulin. Targeted knockout of the mouse insulin receptor gene in β cells, which artificially disrupted or inhibited of β cells insulin signaling pathway, resulted in insulin resistance and secretion dysfunction in β cells[31]. Therefore, the insulin receptor of β cells is indispensable for maintaining the normal function of β cell.

Prolonged exposure of skeletal muscle cells[5], 3T3-L1 adipocytes[32] and cardiac myocytes[33] to palmitate increases ceramide accumulation and inhibition of Akt. Consistent with this, palmitate exposure also caused a reduction of glucose uptake and glycogen synthesis[34], eventually leads to impaired insulin sensitivity. Moreover, high level of free fatty acid (FFA) can disrupt the insulin signaling pathway of islet β cells and peripheral target tissues (liver, adipose tissue and skeletal muscle), leading to signal transduction disorder and furthermore insulin resistance in these tissues[35,36].

Studies have shown that the disturbance of tyrosine phosphorylation of IRS and the abnormal increase of phosphorylation level of serine threonine site block the transmission of insulin signaling and cause cell dysfunction. Vaspin, a member of the serpin family of serine protease inhibitors, is an adipose cytokine with insulin sensitizing effect. Studies have shown that vaspin can inhibit the phosphorylation of insulin receptor and its downstream molecules IRS-1 and IRS-2 in high glucose stimulated vascular smooth muscle cells[37]. In this study, we investigated the effects of vaspin on the expression of IRS-2 mRNA and phosphorylation levels in INS-1 cells. We found that palmitic acid increased serine phosphorylation levels of IRS-2 in INS-1 cells, and reduced the levels of IRS-2 mRNA and total protein. Interestingly, vaspin treatment reversed these effects, indicating that vaspin could inhibit the serine phosphorylation level of IRS-2 in INS-1 cells induced by palmitic acid, further improved the insulin resistance of INS-1 cells.

AKT is a key protein located downstream of PI3K. It plays an important role in regulating cell growth, proliferation and survival, as well as metabolism[38]. Vaspin can act on PI3K/Akt signal pathway of vascular smooth muscle cells, improve insulin resistance and protect free fatty acid mediated apoptosis of vascular smooth muscle cells[39]. Our previous studies also showed that vaspin acted on vascular endothelial cells, enhanced the transduction of PI3K/Akt signaling pathway and improved the insulin resistance of vascular endothelial cells[40]. This study further investigated the effects of vaspin on free fatty acid induced PI3K/Akt insulin signaling pathway in INS-1 cells and whether vaspin can improve the insulin secretion function of islet β cells through this pathway. The results showed that palmitic acid inhibited serine phosphorylation level of Akt in INS-1 cells. Co-treatment with vaspin reversed the inhibition effect of palmitic acid on Akt phosphorylation. In addition, co-administration of PI3K inhibitor ly294002 with vaspin abolished the increase of Akt phosphorylation level induced by vaspin. Together, these observations suggested that vaspin can improve free fatty acids induced insulin resistance in INS-1 cells by regulating the serine phosphorylation of Akt mediated by PI3K. We also found that vaspin can improve the insulin secretion function of islet β cell stimulated by glucose, which could also be reversed after the addition of PI3K inhibitor ly294002. This suggested that vaspin can improve the secretion function of islet β cell by promoting the transduction of PI3K/Akt insulin signaling pathway. Therefore, vaspin can not only improve insulin resistance of islet β cells by promoting PI3K/Akt insulin signaling pathway, but also improve the insulin secretion function of INS-1 cells.

mTOR, which belongs to the phosphatidylinositol related protein kinase family, is a downstream protein of PI3K/AKT signaling pathway. Obesity induced by excess nutrient intake leads to the upregulation of mTORC1/S6K1 signaling in insulin-sensitive tissues, including β cells. The mTOR and its downstream effector ribosomal S6 kinase-1 mediate various biological effects of nutrients, insulin, and energy[41]. Previous study has shown that activation of S6K1 is mediated by leptin in macrophages[42]. When there are excess nutrients in the body, high concentrations of amino acids/glucose are stimulated chronically. It could lead to continuous activation of mTOR/S6K1, resulting in hyperphosphorylation of IRS1-Ser312, IRS1-Ser636/639 or IRS1-Ser307, and inducing insulin resistance[43,44]. In the present study, palmitic acid intervened in INS-1 cells exhibiting high serine phosphorylation levels of IRS-2, significantly inhibited the phosphorylation of mTOR and p70S6K, which could be reversed by vaspin. Interestingly, recent evidence suggests that rapamycin, an inhibitor of mTOR, can block vaspin-mediated signals responsible for insulin resistance. The decrease of IRS/PI3K/Akt signaling pathway may be the direct effect of palmitic acid. Perhaps, interference of the islet signaling pathway is caused by the excessive activation of mTOR/S6K1 signaling pathway induced by palmitic acid.

Akt mediates its proliferative and anti-apoptotic action on cells through several mechanisms. In a recent study by Jia[45], insulin-like growth factor (IGF) treatment of saphenous vein smooth muscle cells induced phosphorylation of PI3K/Akt and promoted the proliferation of the cells. A number of previous studies have also demonstrated that Akt promotes cell growth and proliferation by activating mTOR/p70S6K signaling pathways[4,5]. p70S6K is a direct acting substrate of mTOR. The phosphorylation of p70S6K-thr389 can promote cell proliferation. In this study, the phosphorylation level of p70S6K in PA plus vaspin group was higher than that of PA group, and the CCK-8 experiments also confirmed that vaspin could increase the proliferation of INS-1 cells, and this effect could be inhibited by rapamycin.

Recent studies have suggested that type 2 diabetes may be a chronic inflammatory response mediated by inflammatory factors[46]. A large number of immune cells infiltration and high levels of cytokines were reported to be present in pancreas islets of type 2 diabetes patients[47–49], which caused various degree of damage to pancreatic β cell activity, leading to β cell failure[15–18]. In addition, inhibition of inflammatory cytokine IL-1β can improve dysfunction of human β cell[50]. These studies suggested that islet inflammation is closely associated with type 2 diabetes. Moreover, inflammatory factors have been shown to promote insulin resistance. Recent studies have also shown that insulin receptor signaling pathway and inflammatory cytokines may cross-talk with each other. Inflammatory factors can interfere with insulin signaling pathway of IRS/PI3K/Akt, which is the main molecular mechanism of insulin resistance[51]. Hence, islet inflammation not only damage islet β cells directly, but also cause insulin resistance of islet β cells. In addition to insulin sensitizing, vaspin has also been shown to have anti-inflammatory effect[26,52,53]. A recent study reported that vaspin suppressed the apoptosis and inflammatory factors chemotaxis of high glucose injured vascular smooth muscle cells by inhibiting NF-κB signaling pathway[31]. Vaspin can inhibit the activation of NF-κB induced by TNF-α and the expression of adhesion molecules in human vascular endothelial cells[54]. Our previous study also showed that vaspin can inhibit the activation of NF-κB mediated by proinflammatory factor TNF-α and IL-1 and suppress the expression of the downstream molecules, protecting vascular endothelial cells from inflammatory damage[27]. Free fatty acids can induce islet inflammation and insulin resistance of pancreatic β cells and their interaction can form a vicious cycle, eventually results in β cell dysfunction and even failure[15,48,55,56]. Therefore, we investigated the effect of vaspin on free fatty acid induced NF-κB inflammation signal transduction pathway and whether vaspin can improve the secretion function of islet β cell by inhibiting the transduction of NF-κB inflammatory signaling pathway. The results demonstrated that palmitic acid induced inflammation of islet β cells, while NF-κB inhibitor TPCK inhibited the expression of inflammatory factor NF-κB and enhanced glucose stimulated insulin secretion. Meanwhile, islet β cells pretreated with vaspin also inhibited the expression of NF-κB and enhanced glucose stimulated insulin secretion, which indicated that vaspin promoted the secretion function of pancreatic β cell through NF-κB inflammatory signal pathway. Studies have shown that IKK can not only activate NF-κB by phosphorylating IκB, but also lead to the phosphorylation of IRS-serine 307, which inhibits normal tyrosine phosphorylation[23], and interferes with the insulin signaling pathway, ultimately leads to insulin resistance. The study found that vaspin can inhibit the level of NF-κB in INS-1 cells induced by free fatty acids, indirectly indicating the reduction of activated IKK, and further reduce the inhibition of insulin resistance pathway, thereby improving the function of pancreatic β cells.

This study confirmed that vaspin can relieve insulin resistance of islet β cells through the insulin signaling pathway of IRS-2/PI3K/Akt/mTOR/p70S6K, and inhibit the inflammation of pancreatic β cells through NF-κB signaling pathway, protecting β cells from damage and improving β cell function.

Administration of vaspin on obese CRL: CD-1 (ICR) (ICR) mice fed with high-fat/high-sucrose chow was shown to have improved glucose tolerance and insulin sensitivity reflected by normalized serum glucose level[26]. Rats in this study were fed with high fat diet for 16 weeks and then received vaspin for 4 weeks. We found that vaspin significantly reduced blood glucose level induced by HFD, but had no significant effect on body weight. We speculated that lard oil might have affected the taste or the high fat diet might have prolonged gastric emptying of rats, which led to a decrease in food intake, so that the body weight of the HF group rats increased slowly. Meanwhile, compensatory mechanisms such as increased insulin secretion and increased body metabolism could also lead to a slowly increase in blood sugar and body weight. Although intraperitoneal injection of vaspin improves glucose tolerance and lowers blood sugar levels, it did not reduce body weight in a short period of time. In addition, the glucose tolerance test (OGTT) and insulin tolerance test (ITT) showed that vaspin improved glucose tolerance and insulin sensitivity in high fat diet fed rats. Further, hyperglycemic clamp test also revealed that vaspin significantly improved islet β cell function of the high fat diet fed rats. Therefore, in addition to improving insulin sensitivity, vaspin may also play an important role in improving insulin secretion function of islet β cell.

In summary, results from the current study provide an experimental basis for vaspin to be used as a potential insulin-sensitizing and anti-inflammatory agent for islet β cells, bringing new hope for the treatment and prevention of type 2 diabetes.
